# A Novel Boolean Kernels Family for Categorical Data †

**DOI:** 10.3390/e20060444

**Published:** 2018-06-06

**Authors:** Mirko Polato, Ivano Lauriola, Fabio Aiolli

**Affiliations:** 1 Department of Mathematics, University of Padova, via Trieste 63, 35121 Padova, Italy; 2 Fondazione Bruno Kessler, via Sommarive 18, 38123 Trento, Italy

**Keywords:** boolean kernels, kernel methods, SVM, categorical data

## Abstract

Kernel based classifiers, such as SVM, are considered state-of-the-art algorithms and are widely used on many classification tasks. However, this kind of methods are hardly interpretable and for this reason they are often considered as *black-box* models. In this paper, we propose a new family of Boolean kernels for categorical data where features correspond to propositional formulas applied to the input variables. The idea is to create human-readable features to ease the extraction of interpretation rules directly from the embedding space. Experiments on artificial and benchmark datasets show the effectiveness of the proposed family of kernels with respect to established ones, such as RBF, in terms of classification accuracy.

## 1. Introduction

Large-margin kernel machines (e.g., SVM) are recognized state-of-the-art algorithms in machine learning applications. They are broadly applied to several domains, such as text categorization, spam filtering, RNA function prediction, and so on. However, since these methods typically work on an implicitly defined feature space by resorting to the well-known kernel trick, the interpretability of the resulting model is difficult.

This last aspect is often crucial in specific application areas, such as the medical ones, in which the simple predictive answer is not enough. Being this a requirement for the acceptance of these *black-box* models by end users, in the last decade, several methods have been introduced for rule extraction from SVMs (see [1] for a recent survey). The majority of the proposed approaches try to extract *if–then* rules over the input variables and this task is generally hard when common kernels, e.g., the polynomial and RBF ones, are used.

On the other hand, Decision Trees (DT), thanks to their easy logical interpretation, are very appreciated, especially by non-expert users. The shortcoming of DTs is that, in general, they are not as accurate as more complex methods. In the case of binary valued input data, an alternative approach to make SVM more interpretable consists in defining features that are easy to interpret, for example, features that are propositional (i.e., logical) formulas applied to the input vectors. In particular, Boolean kernels are kernel functions in which the binary input vectors are mapped into an embedding space formed by propositional formulas of the input variables, and, in such space, the dot product is performed.

More formally, a Boolean kernel function $\kappa :{\left\{0,1\right\}}^{n}\times {\left\{0,1\right\}}^{n}\to N$ is defined as κ(x,z)=〈ϕ(x),ϕ(z)〉 where $\phi :{\left\{0,1\right\}}^{n}\to {\left\{0,1\right\}}^{N}$ is the embedding Boolean function with $x,z\in {\left\{0,1\right\}}^{n}$. When the input space is binary, the linear kernel can be seen as a limit case of a Boolean kernel in which the features simply represent the Boolean literals themselves.

In this paper, we propose a new family of Boolean kernels able to produce feature spaces composed by arbitrarily complex propositional formulas. In particular, we first introduce the basic Boolean kernels [2], namely the conjunctive kernel and the disjunctive kernel, for both the monotone and non-monotone case. On top of these kernels, we then propose more complex kernels such as the Disjunctive Normal Form kernel and the Conjunctive Normal Form kernel. For all the proposed kernels, an efficient method to compute them is provided. We assess the quality of the proposed kernels in terms of classification accuracy on several categorical datasets, and compare their performance against state-of-the-art kernels, such as the RBF kernel, and other Boolean kernels proposed in the literature.

The reminder of this paper is structured as follows: in Section 2, we give an overview of the existing work related to Boolean kernels. In Section 3, we present the proposed Boolean kernels family and, in Section 4, we discuss their computational complexity. Finally, Section 5 shows all the performed experiments on several benchmark categorical datasets.

## 2. Related Work

Sadohara [3] was the first to introduce the idea of Boolean kernel. In that work, the concept of Boolean kernel is actually related to a single kernel called DNF kernel. Specifically, he proposed a SVM for learning Boolean functions: since every Boolean (i.e., logical) function can be expressed in terms of Disjunctive Normal Form (DNF) formulas, the proposed kernel creates a feature space containing all possible conjunctions of negated or non-negated Boolean variables.

For instance, the feature space for a two variables, e.g., $x_{1},x_{2}$, DNF contains the following $3^{2}-1$ features: $x_{1},x_{2},\neg x_{1},\neg x_{2},x_{1}\wedge x_{2},x_{1}\wedge \neg x_{2},\neg x_{1}\wedge x_{2},\neg x_{1}\wedge \neg x_{2}$. The resulting decision function of a kernel machine which employs the DNF kernel can be represented as a weighted linear sum of conjunctions (Representer Theorem [4,5]), which in turn can be seen as a kind of “soft” DNF.

Formally, the DNF kernel between $x,z\in R^{n}$ is defined as

$$\kappa_{\mathrm{dnf}}\left(x,z\right)=-1+\prod_{i=1}^{n}\left(2x_{i}z_{i}-x_{i}-z_{i}+2\right),$$

while its monotone (i.e., without negations) form is the following

$$\kappa_{\mathrm{mdnf}}\left(x,z\right)=-1+\prod_{i=1}^{n}\left(x_{i}z_{i}+1\right).$$

By restricting the domain of the vectors in ${\left\{0,1\right\}}^{n}$, the computation of the kernels is simplified as follows

$$\kappa_{\mathrm{dnf}}\left(x,z\right)=-1+2^{\langle x,z\rangle +\langle \bar{x},\bar{z}\rangle },\;\kappa_{\mathrm{mdnf}}\left(x,z\right)=-1+2^{\langle x,z\rangle },$$

where $\bar{x}=1_{n}-x$ is the vector with all the binary entries swapped. Note that the sum $\langle x,z\rangle +\langle \bar{x},\bar{z}\rangle$ simply counts the number of common “bits” between x and z. These last two kernels were independently discovered in [6,7].

A drawback of this type of kernels is the exponential growth of the size of the feature space with respect to the number of involved variables, i.e., $3^{n}-1$ for *n* variables. To give the possibility of controlling the size of the feature space, Sadohara et al. [8] proposed a variation of the DNF kernel in which only conjunctions with up to *d* variables (i.e., *d*-ary conjunctions) are considered. Over binary vectors, this kernel, dubbed d-DNF kernel, is defined as

$$\kappa_{\mathrm{dnf}}^{d}\left(x,z\right)=\sum_{i=1}^{d}\left(\begin{array}{c} \langle x,z\rangle +\langle \bar{x},\bar{z}\rangle \\ i \end{array}\right),$$

and trivially, if d=n, $\kappa_{\mathrm{dnf}}^{d}\left(x,z\right)=\kappa_{\mathrm{dnf}}\left(x,z\right)$. A nice property of the d-DNF kernel is that it yields a nested sequence of hypothesis spaces, i.e., $H_{1}\subseteq H_{2}\subseteq \ldots \subseteq H_{n}$. Thus, choosing a degree *d* (also known as “arity”) for the kernel implicitly means controlling the capacity of the hypothesis space, which is a very important aspect in learning. The same “trick” can be applied to the monotone d-DNF kernel [9]:

$$\kappa_{\mathrm{mdnf}}^{d}\left(x,z\right)=\sum_{i=1}^{d}\left(\begin{array}{c} \langle x,z\rangle \\ i \end{array}\right).$$

Instead of limiting the number of involved variables, Zhang et al. [10] proposed a parametric version of the DNF and mDNF kernel. Specifically, given $x,z\in {\left\{0,1\right\}}^{n}$ and σ>0, then

$$\begin{array}{cc} \kappa_{\mathrm{dnf}}^{\left(\sigma \right)}\left(x,z\right) & =-1+\prod_{i=1}^{n}\left(\sigma x_{i}z_{i}+\sigma \left(1-x_{i}\right)+\sigma \left(1-z_{i}\right)+1\right),\\ \kappa_{\mathrm{mdnf}}^{\left(\sigma \right)}\left(x,z\right) & =-1+\prod_{i=1}^{n}\left(\sigma x_{i}z_{i}+1\right). \end{array}$$

The parameter σ induces an inductive bias towards simpler or more complex DNF formulas. Specifically, for σ in the range [0,1] a bias towards shorter DNF is given, while for σ>1 the bias is more towards longer DNF. When σ=1, then $\kappa_{\mathrm{dnf}}^{\left(\sigma \right)}\left(x,z\right)=\kappa_{\mathrm{dnf}}\left(x,z\right)$, and the same for the monotone DNF kernel. In the following, we refer to these kernel as σ-DNF and σ-mDNF kernel, respectively. Zhang et al. also proved that, for binary input vectors, the polynomial kernel, i.e., $\kappa_{\mathrm{POLY}}^{p}\left(x,z\right)={\left(\sigma \langle x,z\rangle +c\right)}^{p}$, $c\in R_{+}$, is a Boolean kernel, even though they did not provide any formal definition of Boolean kernel. Nonetheless, an important observation is that the embedding space of a polynomial kernel is composed by all the monomials (that are conjunctions) up to the degree *p*. Thus, the only difference between the polynomial one and the d-DNF kernel are the weights associated to the features. It is also noteworthy that, in the binary case, the embedding of the polynomial kernel contains sets of equivalent features, e.g., for p=4 and $x\in {\left\{0,1\right\}}^{2}$, the value of the features (in the feature space) $x_{1}^{3}x_{2},x_{1}^{2}x_{2}^{2},x_{1}x_{2}^{3}$ are equivalent to the feature $x_{1}x_{2}$.

A kernel related to the polynomial is the all-subset kernel [11,12], defined as

$$\kappa_{\subseteq }\left(x,z\right)=\prod_{i=1}^{n}\left(x_{i}z_{i}+1\right),$$

which considers a space with a feature for each subset of the input variables, including the empty subset. It is different from the polynomial because it does not limit the number of considered monomials, and it gives the same weight to all the features. It is easy to see that the all-subset kernel and the monotone DNF kernel are actually the same kernel up to the constant −1, i.e., $\kappa_{\subseteq }\left(x,z\right)=\kappa_{\mathrm{mdnf}}\left(x,z\right)+1$.

Both the polynomial and the all-subsets kernel have limited control of which features they use and how they are weighted. The polynomial kernel uses only monomials of degree up to *p* with a weighting scheme depending on a parameter (*c*). The all-subsets, instead, makes use of the monomials corresponding to all possible subsets of the *n* input variables.

A restricted version of the all-subset kernel is the ANOVA kernel [11] in which the embedding space is formed by monomials with a fixed degree *d* without repetition. For example, given $x\in {\left\{0,1\right\}}^{3}$ the feature space of the all-subset kernel would be made by the features $x_{1},x_{2},x_{3},x_{1}x_{2},x_{1}x_{3},x_{2}x_{3},x_{1}x_{2}x_{3}$ and ∅, while for the ANOVA kernel of degree 2 it would be composed by $x_{1}x_{2},x_{1}x_{3}$ and $x_{2}x_{3}$. Formally, the ANOVA kernel is defined as follows

$$\kappa_{A}^{d}\left(x,z\right)=\sum_{1\leq i_{1}<i_{2}<\ldots <i_{d}\leq n}\;\prod_{j=1}^{d}x_{i_{j}}z_{i_{j}},$$

where $i_{1},i_{2},\ldots ,i_{d}$ are all the possible sets of indices of cardinality *d*, taken from the set {1,…,n}.

In [13,14], Boolean kernels are used for studying the learnability of logical formulas, specifically DNF formulas, using maximum margin algorithms, such as SVM. In particular, in [14], the authors showed the learning limitations of some Boolean kernels inside the PAC (Probably Approximately Correct) framework. Moreover, in [13], Kowalczyk at al. proposed a generalization of the Sadohara mDNF kernel. A special case of this kernel is represented by the σ-mDNF kernel.

From the application point of view, Boolean kernels have been successfully applied on many learning tasks, such as face recognition [15,16], spam filtering [17], load forecasting [18], and generic binary classification tasks [8,10].

## 3. A New Family of Boolean Kernels

In this section, we propose a new family of Boolean kernels which owns the characteristic of creating feature spaces that are very easy to understand, since they are based on logic. Specifically, features are logical formulas (of a fixed form) over the input Boolean variables.

Firstly, we present the most basic Boolean kernel and then, for both the *monotone* and the *non-monotone* cases, we propose kernels which mimic the conjunctive operator (*and*) and the disjunctive operator (*or*). Then, we provide an efficient way to combine these “base” Boolean kernels to obtain more complex ones, such as kernels with feature spaces composed by normal form formulas.

Throughout the paper, unless specified otherwise, we refer to vectors $x,z\in {\left\{0,1\right\}}^{n}$ as binary (Boolean) vectors of dimension $n\in N_{+}$. We also use $X\equiv \{i\;|\;x_{i}=1\}$ and $Z\equiv \{i\;|\;z_{i}=1\}$ as the sets interpretation of those vectors, while the set U≡{1,…,n} indicates the universal set. Given a set A, we refer to its *i*-th element with $A_{i}$ for some enumeration of the elements of A. With the notation ${\left[A\right]}^{k}\equiv \left\{S\subseteq A||S|=k\right\}$, we express the set of all the subsets of A of cardinality *k*. It is worth noticing that, for any binary vector x, ${∥x∥}_{2}^{2}={∥x∥}_{1}$ holds, which is the number of ones contained in it. For the sake of brevity, we refer to this quantity with |x|. Moreover, $1_{n}$ denotes the *n*-dimensional vector with all entries equal to 1, and with the notation 〈·,·〉 we refer to the dot product. The symbol ⊙ denotes the entry-wise multiplication between matrices. Finally, given a function $f:X\to Y^{n}$, Y⊆R, then |f| denotes the dimension of its codomain, i.e., *n*.

For each of the proposed kernels, the embedding function is provided in the general form $\phi :x\mapsto \phi^{\left(b\right)}{\left(x\right)}_{b\in B}$, where B is a set of Boolean functions (formulas) over variables of x such that $\phi^{\left(b\right)}\left(x\right)=b\left(x\right)$ is a truth value associated with the application of the formula *b* to x. For example, let $b\left(x\right)=x_{1}\wedge x_{2}$ and v=[0,1,0,1], then b(v)=0, that is *false*. For the sake of simplicity, for each new kernel, only the set B from which the Boolean formulas are taken is defined.

### 3.1. Monotone Boolean Kernels

A Boolean function (or formula) $f:{\left\{0,1\right\}}^{n}\to \left\{0,1\right\}$ is called *monotone* if replacing a 0 (i.e., *false*) with a 1 (i.e., *true*) in the input can only increase *f*’s value, i.e., the truth value can only change from *false* to *true*. In other words, a formula *f* is *monotone* if it does not have any *not* operator.

#### 3.1.1. Monotone Literal Kernel

In logic, a literal is an atomic formula or its negation. Here, we are in the monotone setting, so we refer to a literal only in its positive form. In this case, the embedding is formed by Boolean literals taken from the set $B\equiv \{f_{i}|f_{i}\left(x\right)=x_{i}\}$. Hence, the monotone Literal (mL) kernel, $\kappa_{\mathrm{mL}}\left(x,z\right)$, will count how many *true* (i.e., positive) input literals the vectors have in common. Actually, $\kappa_{\mathrm{mL}}$ is exactly the linear kernel $\kappa_{\mathrm{LIN}}\left(x,z\right)=\langle x,z\rangle$, which simply performs the dot product between the two input binary vectors.

#### 3.1.2. Monotone Conjunctive Kernel

In Boolean algebra, given two Boolean variables x,z∈{0,1}, the conjunction (i.e., *and*) between *x* and *z*, denoted by x∧z, is satisfied if and only if x=z=1, that is if and only if both variables are *true*. Given two vectors $x,z\in {\left\{0,1\right\}}^{n}$, the monotone Conjunctive (mC) kernel [19] $\kappa_{\mathrm{mC}}^{c}\left(x,z\right)$ counts how many monotone conjunctions of the input variables, of a fixed arity *c*, are satisfied in both x and z. In particular, the embedding is defined by Boolean formulas taken from $B\equiv \{f_{i}|f_{i}\left(x\right)={⋀}_{j\in {\left[U\right]}_{i}^{c}}x_{j}\}$, which represent all the possible conjunctions of *c* literals (i.e., variables) taken from x. The dimension of the resulting feature space is nc, that is the number of all the combinations of *c* different variables taken from the input *n*-dimensional space. To count all the possible conjunctions of *c* variables satisfied in both x and z, we need to calculate the number of combinations of *c* monomials that can be formed by using all the positive variables in both the vectors, that is the value of the kernel $\kappa_{\mathrm{mL}}\left(x,z\right)$. Hence, we obtain:

$$\kappa_{\mathrm{mC}}^{c}\left(x,z\right)=\left(\begin{array}{c} \kappa_{\mathrm{mL}}\left(x,z\right)\\ c \end{array}\right)=\left(\begin{array}{c} \langle x,z\rangle \\ c \end{array}\right).$$

It is easy to see that for binary vectors $\kappa_{\mathrm{mC}}^{c}$ is actually the ANOVA kernel of degree *c* [11].

As shown in [19], we can express the Sadohara mDNF Kernel [3] as a linear combination of mC-kernels of arity 1≤d≤n as in the following:

$$\kappa_{\mathrm{mdnf}}\left(x,z\right)=2^{\langle x,z\rangle }-1=\sum_{d=0}^{n}\left(\begin{array}{c} \langle x,z\rangle \\ d \end{array}\right)-1=\sum_{d=1}^{n}\kappa_{\mathrm{mC}}^{d}\left(x,z\right).$$

A similar construction also holds for the d-mDNF kernel.

#### 3.1.3. Monotone Disjunctive Kernel

The disjunction of two Boolean variables x,z∈{0,1}, denoted by x∨z, is not satisfied if and only if x=z=0, that is if and only if both variables are *false*, or, in other words, it is satisfied anytime at least one of the variables is *true*. The monotone Disjunctive (mD) kernel [19], $\kappa_{\mathrm{mD}}^{d}\left(x,z\right)$ counts how many monotone disjunctions, of a fixed arity *d*, are satisfied in both x and z. The embedding of this kernel is defined by Boolean formulas taken from $B\equiv \{f_{i}|f_{i}\left(x\right)={⋁}_{j\in {\left[U\right]}_{i}^{c}}x_{j}\}$, with a feature space of dimension nd. To explain how to count the common positive disjunctions in both x and z, we can rely on the analogy between binary vectors and sets. An active disjunction of *d* literals for X can be defined as a set of *d* elements taken from the universe U, let us call it $U_{d}$, such that $\exists \;a\in U_{d}\;|\;a\in X$. Anytime $\exists \;a,b\in U_{d}\;|\;a\in X\wedge b\in Z$ (potentially a=b), then $U_{d}$ is an *active subset* for X and Z. Using this interpretation, we can say that the value of the kernel is the number of active subsets $U_{d}$ in common between X and Z. We can count the number of these subsets in a negative fashion. Starting from the number of all possible subsets, which is (|U|d), we remove the inactive subsets for X and for Z. An inactive subset for X is a set such that it does not contain any element of X, and the number of this kind of sets is (|U∖X|d). Analogously, we can do the same for Z. Now, we have removed twice the subsets formed by elements taken from $\bar{X\cup Z}\equiv U\setminus \left(X\cup Z\right)$ and hence we need to add its contribution once, that is (|U∖(X∪Z)|d). We can now define $\kappa_{\mathrm{mD}}^{d}$ as

(1) κmDd(x,z)=|U|d−|U∖X|d−|U∖Z|d+|U∖(X∪Z)|d=nd−n−〈x,x〉d−n−〈z,z〉d+n−〈x,x〉−〈z,z〉+〈x,z〉d.

### 3.2. Non-Monotone Boolean Kernels

Converse to the monotone case, *non-monotone* Boolean formulas can contain negated variables, e.g., $\neg x_{i}$, thus the mL-kernel is not expressive enough to be the simplest non-monotone Boolean kernel because it does not consider negated variables.

#### 3.2.1. Non-Monotone Literal Kernel

To include the contribution of the negated variables, we need to add the number of *false* variables in common between x and z to the mL-kernel. This can be calculated by the *negation* kernel, defined as

$$\begin{array}{cc} \kappa_{\mathrm{NEG}}\left(x,z\right) & =\langle \left(1_{n}-x\right),\left(1_{n}-z\right)\rangle \\ & =n-\langle x,x\rangle -\langle z,z\rangle +\langle x,z\rangle , \end{array}$$

in which the embedding is defined by Boolean functions taken from $B\equiv \{f_{i}|f_{i}\left(x\right)=\neg x_{i}\}$. Hence, the non-monotone Literal (L) kernel, $\kappa_{L}\left(x,z\right)$, counts how many *true* and *false* variables x and z have in common, and it is defined as a sum of kernels [11] as in the following:

$$\begin{array}{cc} \kappa_{L}\left(x,z\right) & =\kappa_{\mathrm{mL}}\left(x,z\right)+\kappa_{\mathrm{NEG}}\left(x,z\right)\\ & =n-\langle x,x\rangle -\langle z,z\rangle +2\langle x,z\rangle . \end{array}$$

#### 3.2.2. Non-Monotone Conjunctive Kernel

The non-monotone Conjunctive (C) kernel counts how many *non-monotone* conjunctions of a certain arity *c* are satisfied in both x and z. The embedding is defined by boolean functions in the set B of all the non-monotone conjunctions of *c* literals. Formally, given the set $U_{c}^{*}\equiv \left\{S\subseteq \left\{1,\ldots ,2n\right\}||S|=c,∄i,j\in S\;s.t.\;i=2j\right\}$ and the function $g\left(x,i\right)=x_{i}$ if i≤n or $g\left(x,i\right)=\neg x_{i}$ otherwise, then $B\equiv \{f_{i}|f_{i}\left(x\right)={⋀}_{j\in {\left[U_{c}^{*}\right]}_{i}}g\left(x,j\right)\}$. Since we are considering conjunctions of variables, this kernel will count how many combinations of (possibly negated) common variables there are between x and z. Thus, relying on these considerations and on the definition of $\kappa_{L}$, we can finally define the $\kappa_{C}^{c}\left(x,z\right)$ as follows:

κCc(x,z)=κL(x,z)c=n−〈x,x〉−〈z,z〉+2〈x,z〉c.

Similar to the monotone case, we can express the Sadohara DNF Kernel [3] as a linear combination of C-kernels of arity 1≤d≤n as in the following:

κdnf(x,z)=2〈x,z〉+〈x¯,z¯〉−1=∑i=0n〈x,z〉+〈x¯,z¯〉i−1=∑i=0nκmL(x,z)+κNEG(x,z)i−1=∑i=0nκL(x,z)d−1=∑i=1nκCd(x,z).

An analogous construction also holds for the d-DNF kernel.

#### 3.2.3. Non-Monotone Disjunctive Kernel

The non-monotone Disjunctive (D) kernel counts how many *non-monotone* disjunctions, of a certain arity *d*, are satisfied in both x and z. The embedding is defined by Boolean formulas in the set B of all the non-monotone disjunctions of *d* literals. Formally, $B\equiv \{f_{i}|f_{i}\left(x\right)={⋁}_{j\in {\left[U_{d}^{*}\right]}_{i}}g\left(x,j\right)\}$, with $U_{d}^{*}$ and *g* defined as in the previous section. As for the *monotone* case, we derive the kernel function in a negative fashion. The number of every possible combination of arity *d* of variables that can be also in their negated form is 2dnd. For both x and z, we have to discard the combinations that are *false*, which are exactly nd because for each set of *d* different variables, there exists only one assignment of the negations such that the disjunction is *false*. For example, given the variables $x_{1}=1,x_{2}=0$ and $x_{3}=1$, only the disjunction $\neg x_{1}\vee x_{2}\vee \neg x_{3}$ is *false* and all the others $2^{3}-1$ negation assignments are *true*. Then, we have to re-add the *false* combinations that have been discarded twice, that is the combinations made with variables that are *false* in both the vectors. This can be seen as the opposite of what C-kernel computes, but, since we generate all the possible combinations with all the possible negation assignments, the counting is actually the same as the C-kernel. Finally, we can define the $\kappa_{D}^{d}\left(x,z\right)$ as

κDd(x,z)=(2d−2)nd+κCd(x,z)

### 3.3. Boolean Kernels Combination

Given the Boolean kernels defined in the previous sections, we have now all the basic elements to build new Boolean kernels that represent a specific Boolean concept. Table 1 shows a summary of all the presented Boolean kernels. It is easy to see that all the kernels are function of dot products of the input vectors, and this allows us to create new kernels by replacing those dot products with other Boolean kernels. The logical interpretation of the new kernel depends on how the base kernels are combined. In the following, we present some new Boolean kernels generated by using the above mentioned method.

#### 3.3.1. DNF Kernels

A disjunctive normal form (DNF) is a normalization of a logical formula which is a disjunction of conjunctive clauses. In the monotone case, both conjunctive and disjunctive clauses are monotone, i.e., the literals are only in their positive form. Since DNFs are disjunctions of conjunctive clauses, we can combine the embedding maps of the mC-kernel and the mD-kernel in this way $\phi_{\mathrm{mDNF}}^{d,c}:x\mapsto \phi_{\mathrm{mD}}^{d}\left(\phi_{\mathrm{mC}}^{c}\left(x\right)\right),$ obtaining the desired feature space for the monotone DNF, which leads to the definition of the mDNF-kernel as in the following:

κmDNFd,c(x,z)=ncd−nc−κmCc(x,x)d−nc−κmCc(z,z)d+nc−κmCc(x,x)−κmCc(z,z)+κmCc(x,z)d.

Note that we need to know the dimension of the feature space of the mC-kernel. The features of this kernel are actually monotone DNF formulas with exactly *d* disjunctions of conjunctive clauses of arity *c*. In the non-monotone case, we proceed in a similar way but, since the conjunctive clauses are non-monotone, we have to use the C-kernel instead of the mC-kernel. So, the feature space can be created by composing the embedding map of the mD-kernel with the one of the C-kernel, that is, $\phi_{\mathrm{DNF}}^{d,c}:x\mapsto \phi_{\mathrm{mD}}^{d}\left(\phi_{C}^{c}\left(x\right)\right).$ Consequently, the DNF-kernel is defined as follows:

κDNFd,c(x,z)=2cncd−2cnc−κCc(x,x)d−2cnc−κCc(z,z)d+2cnc−κCc(x,x)−κCc(z,z)+κCc(x,z)d.

#### 3.3.2. CNF Kernels

A logic formula is in conjunctive normal form (CNF) if it is composed of conjunctions of disjunctive clauses. Clearly, in its monotone form it does not contain any negated literal. By using a similar approach as for the mDNF-kernel, the feature space is defined by the function $\phi_{\mathrm{mCNF}}^{d,c}:x\mapsto \phi_{\mathrm{mC}}^{d}\left(\phi_{\mathrm{mD}}^{c}\left(x\right)\right),$ and hence in the kernel function we replace the dot products inside the mC-kernel with the mD-kernel as in the following:

κmCNFd,c(x,z)=κmDd(x,z)c.

The resulting feature space is composed of monotone CNF formulas with exactly *c* conjunctions of disjunctive clauses of arity *d*. By swapping the mD-kernel with the D-kernel, we can easily obtain the non-monotone CNF kernel:

κCNF(x,z)=κDd(x,z)c,

which has associated the embedding function $\phi_{\mathrm{CNF}}^{d,c}:x\mapsto \phi_{\mathrm{mC}}^{d}\left(\phi_{D}^{c}\left(x\right)\right).$

For the sake of brevity, in the rest of the paper, we indicate the mDNF-kernel having *d* disjunctions and *c* conjunctions with either the notation mDNF(*d*,*c*)-kernel or simply mDNF(*d*,*c*).

## 4. Computational Complexity

The computational complexity of the Boolean kernels described in the previous sections is bounded by the complexity of the calculation of the binomial coefficient, that is O(k) with *k* the arity of the combinations. Hence, computing an entire kernel matrix over *n*-dimensional examples taken from a dataset with *l* examples would lead to a complexity of $O(\left(k+n\right)\cdot l^{2})$. Even though it is not possible to reduce such complexity, we can take advantage of the recursive nature of the binomial coefficient to compute the kernels in a recursive fashion. By doing so, it is possible to compute higher degree kernel matrices by leveraging on kernel matrices of lower degrees.

Let the matrix $K^{0}$ be the base (Boolean) kernel matrix over the dataset D, such that $K_{i,j}^{0}=\langle \phi \left(x_{i}\right),\phi \left(x_{j}\right)\rangle$, for $x_{i},x_{j}\in D$ and some ϕ with codomain ${\left\{0,1\right\}}^{n}$. Then, we can recursively define the Boolean kernels for both monotone and the non-monotone case as described in the following.

### 4.1. mC-Kernel

By definition, the mC-kernel matrix of arity 1, that is $K_{\mathrm{mC}}^{1}$, is equivalent to the base kernel matrix $K^{0}$. By using $K^{0}$ as base case, we can recursively define the mC-kernel matrix as

$$K_{\mathrm{mC}}^{c+1}=K_{\mathrm{mC}}^{c}⊙\left(\frac{1}{c+1}\left(K_{\mathrm{mC}}^{1}-c1_{l}1_{l}^{⊺}\right)\right).$$

### 4.2. mD-Kernel

Let us define the matrices

$$S=\mathrm{diag}\left(K^{0}\right)\cdot 1_{l}^{⊺},\;N_{x}=n1_{l}1_{l}^{⊺}-S,\;\;\mathrm{and}\;\;N_{xz}=N_{x}-S^{⊺}+K^{0}.$$

Then, we define, recursively in its parts, the mD-kernel matrix $K_{\mathrm{mD}}^{d}$ as

$$K_{\mathrm{mD}}^{d}=N^{\left(d\right)}-N_{x}^{\left(d\right)}-{N_{x}^{\left(d\right)}}^{⊺}+N_{xz}^{\left(d\right)},$$

where

$$\begin{array}{cc} N^{\left(d+1\right)} & =N^{\left(d\right)}⊙\left(\frac{n-d}{d+1}1_{l}1_{l}^{⊺}\right),\\ N_{x}^{\left(d+1\right)} & =N_{x}^{\left(d\right)}⊙\left(\frac{1}{d+1}\left(N_{x}-d1_{l}1_{l}^{⊺}\right)\right),\;\mathrm{and}\;\\ N_{xz}^{\left(d+1\right)} & =N_{xz}^{\left(d\right)}⊙\left(\frac{1}{d+1}\left(N_{xz}-d1_{l}1_{l}^{⊺}\right)\right), \end{array}$$

with the corresponding base cases $N^{\left(1\right)}=n1_{l}1_{l}^{⊺}$, $N_{x}^{\left(1\right)}=N_{x}$ and $N_{xz}^{\left(1\right)}=N_{xz}$.

### 4.3. C-Kernel

By relying on the previous definition of S and the base case kernel matrix

$$K_{C}^{1}=n1_{l}1_{l}^{⊺}-S-S^{⊺}+2K^{0},$$

the C-kernel matrix can be recursively defined by

$$K_{C}^{c+1}=K_{C}^{c}⊙\left(\frac{1}{c+1}\left(K_{C}^{1}-c1_{l}1_{l}^{⊺}\right)\right).$$

### 4.4. D-Kernel

Using the previous definitions of the matrices $N^{\left(d\right)}$ and $K_{C}^{c}$, we can define the D-kernel matrix, recursively in its parts, as

$$K_{D}^{d+1}=\left(\left(2^{d}-2\right)1_{l}1_{l}^{⊺}\right)⊙N^{\left(d\right)}-K_{C}^{d}.$$

Both the standard and the recursive definition have been implemented in a freely available Python module *pyros* available at the following URL: https://github.com/makgyver/pyros.

## 5. Evaluation

### 5.1. Evaluation Protocol

In all experiments, the kernels have been normalized using the well-known formula

$$\tilde{\kappa }\left(x,z\right)=\frac{\kappa (x,z)}{\sqrt{\kappa (x,x)\kappa (z,z)}}.$$

We assessed the proposed Boolean kernels by using SVM as kernel machine and we compared them with the linear kernel, the RBF kernel, the (monotone) DNF kernel proposed by Sadohara et al. [3], the d-DNF kernel [9], the σ-mDNF kernel [10] and the Tanimoto kernel.

Both validation and test were evaluated in terms of the Area Under the ROC Curve (AUC). The used validation method is a five-fold nested cross validation. For each dataset, the test was repeated 10 times and the average performances were recorded. Specifically, we validated the misclassification cost parameter *C* for the SVM in the set $\left\{2^{-4},2^{-3},\ldots ,2^{4}\right\}$; for each of the proposed kernels we validated both the conjunctive arity *c* (when applicable) and the disjunctive arity *d* (when applicable) in the set {1,…,5}, for the RBF kernel we validated the hyper-paramater $\gamma \in \{10^{-4},\ldots ,10^{4}\}$, while for the *d*-mDNF we fixed d=5. Finally, for the σ-mDNF kernel we validated σ in the set {0.2,0.5,1,2}. All the experiments were implemented in Python using the machine learning module Scikit-learn [20]. The source code is freely available at https://github.com/makgyver/pyros.

The benchmark datasets used for the experiments are reported in Table 2. These datasets are freely available from the UCI repository [21] and the KEEL repository [22]. We selected datasets with binary or categorical features and for each of them the following preprocessing steps were performed:

- Instances with missing attributes were removed.
- categorical features, including the binary ones, were mapped into binary features by means of the *one-hot* encoding [23]. This preprocessing keeps for every example in the dataset the same number of ones , or in other words every input vector has the same euclidean norm.
- Non-binary tasks were artificially transformed into binary ones, by arranging the classes into two groups while trying to keep the number of instances balanced.

### 5.2. Experimental Results

The first set of experiments assessed the quality of the proposed kernels. The average AUCs over 10 runs as well as the standard deviations are reported in Table 3. The last row of each table summarizes the average rank achieved by all kernels over the benchmark datasets.

It is evident from the tables that normal form (NF) kernels perform on average better than the other Boolean kernels, with the only exception of the C-kernel on the AUC metric. This is reasonable since normal form kernels are a generalization of the (m)C-kernel and the (m)D-kernel. However, after the validation procedure, there is no guarantee that a NF kernel is always better than their correspondent base kernels, as underlined by the very good performance of the C-kernel. Another interesting observation is that both the D-kernel and the mD-kernel are almost always the worst performing ones, and this can be explained by the fact that they are less expressive than the competing kernels.

Since NF kernels have shown very good performances, we built, for each normal form, the average kernel over all the degrees, that is:

$$\kappa_{\mathrm{avg}.\mathrm{NF}}=\frac{1}{C\times D}\sum_{d=1}^{D}\sum_{c=1}^{C}\kappa_{NF}^{d,c}\left(x,z\right).$$

In this way, given a normal form, the feature space of the resulting kernel contains all the normal form formulas for each of the degrees (c,d) with 1≤c≤C and 1≤d≤D. Since we have fixed the maximum degrees *C* and *D* or all the kernels, and the $\kappa_{\mathrm{avg}.\mathrm{NF}}$ has no other hyper-parameters, it did not require any further validation.

The average AUCs over the 10 runs as well as the standard deviations are reported in Table 4.

In general, we can see that the normal form kernels seem to achieve performances almost always comparable or higher than the competing kernels. Good performance is also achieved by the d-mDNF kernel which we have shown is the summation of *d* mC-kernels.

An interesting observation can be done regarding the monks-1 dataset which can be explained by a mDNF rule. The obtaining results on monks-1 show that most of the proposed Boolean kernels achieve an AUC of 100% while all other kernels are not able to achieve this perfect score. This is because these Boolean kernels contain the target formula, or a set of related formulas, in the feature space.

All the reported results are further confirmed by other experiments with other metrics, such as precision, recall and F1, however they are not reported here for space reasons.

## 6. Conclusions

We present a family of Boolean kernels designed to have feature spaces composed by logical formulas that can be exploited to interpret the solution of a large-margin kernel machine, such as an SVM. For all kernels, we provide the logic interpretation and an efficient way to compute them. Experimental results on many categorical datasets show that the presented kernels achieve a performance comparable to the performance of state-of-the-art kernels (such as the RBF) and other Boolean kernels. In particular, we observed that, in general, those kernels corresponding to normal form achieve very good performances. In the future, we aim to develop methods able to learn how to combine different Boolean kernels, for example by means of Multiple Kernel Learning algorithms. Moreover, we also aim to find efficient and effective ways to interpret the solution of SVMs based on Boolean kernels. 

## Figures and Tables

**Table 1:** Summary of the just presented Boolean kernels: $x,z\in {\left\{0,1\right\}}^{n}$ and |ϕ| stands for the dimension of the feature space.

κ	κ(x,z)	κ(x,x)	|ϕ|
$\kappa_{\mathrm{mL}}$	〈x,z〉	|x|	*n*
$\kappa_{\mathrm{mC}}^{c}$	κmL(x,z)c	|x|c	nc
$\kappa_{\mathrm{mD}}^{d}$	nd−n−|x|d−n−|z|d+n−|x|−|z|+〈x,z〉d	nd−n−|x|d	nd
$\kappa_{\mathrm{NEG}}$	n−|x|−|z|+〈x,z〉	n−|x|	*n*
$\kappa_{L}$	$\kappa_{\mathrm{mL}}+\kappa_{\mathrm{NEG}}$	*n*	2n
$\kappa_{C}^{c}$	κL(x,z)c	nc	2cnc
$\kappa_{D}^{d}$	(2d−2)nd+κCd(x,z)	(2d−1)nd	2dnd

**Table 2:** Datasets information: name, number of instances (# Examples), number of features (# Features), class distribution and number of active variables for example.

Dataset Name	# Examples	pos/neg (%)	# Features	$m={∥x∥}_{1}$
zoo	101	40/60	36	16
promoters	106	50/50	228	57
lymphography	148	45/55	44	15
house-votes	232	46/54	32	16
soybean	266	54/46	97	35
spect	267	79/21	44	22
breast	277	71/29	41	9
primary-tumor	339	41/59	34	15
monks-1	432	50/50	17	6
crx	653	55/45	40	9
tic-tac-toe	958	65/35	27	9
flare	1066	49/51	41	11
car	1728	30/70	21	6
dna_bin	2000	47/53	180	47
splice	3175	48/52	240	60
kr-vs-kp	3196	52/48	73	36

**Table 3:** AUC performances on benchmark datasets. For each dataset the best performing kernel is highlighted with both the **boldface** font and with a dot (·).

Dataset	mC	mD	C	D	mDNF	mCNF	DNF	CNF
breast	$\underset{\pm 1.27}{71.19}$	$\underset{\pm 1.51}{71.68\cdot }$	$\underset{\pm 1.87}{71.52}$	$\underset{\pm 1.80}{71.70}$	$\underset{\pm 1.73}{71.21}$	$\underset{\pm 2.07}{71.31}$	$\underset{\pm 1.82}{71.54}$	$\underset{\pm 1.90}{71.50}$
car_bin	$\underset{\pm 0.00}{100.00\cdot }$	$\underset{\pm 0.08}{99.97}$	$\underset{\pm 0.00}{100.00\cdot }$	$\underset{\pm 0.12}{99.62}$	$\underset{\pm 0.00}{100.00\cdot }$	$\underset{\pm 0.00}{100.00\cdot }$	$\underset{\pm 0.00}{100.00\cdot }$	$\underset{\pm 0.00}{100.00\cdot }$
crx	$\underset{\pm 0.39}{91.77}$	$\underset{\pm 0.37}{92.04}$	$\underset{\pm 0.22}{92.11\cdot }$	$\underset{\pm 0.28}{91.99}$	$\underset{\pm 0.34}{91.81}$	$\underset{\pm 0.38}{91.86}$	$\underset{\pm 0.29}{92.08}$	$\underset{\pm 0.30}{92.03}$
dna_bin	$\underset{\pm 0.05}{99.03}$	$\underset{\pm 0.06}{98.98}$	$\underset{\pm 0.05}{99.07\cdot }$	$\underset{\pm 0.05}{98.96}$	$\underset{\pm 0.05}{99.07\cdot }$	$\underset{\pm 0.04}{99.01}$	$\underset{\pm 0.05}{99.07\cdot }$	$\underset{\pm 0.05}{99.06}$
flare_bin	$\underset{\pm 0.13}{94.80}$	$\underset{\pm 0.13}{94.91}$	$\underset{\pm 0.13}{94.91}$	$\underset{\pm 0.10}{94.91}$	$\underset{\pm 0.11}{94.88}$	$\underset{\pm 0.11}{94.89}$	$\underset{\pm 0.10}{94.92\cdot }$	$\underset{\pm 0.13}{94.90}$
house-votes-84	$\underset{\pm 0.26}{99.23}$	$\underset{\pm 0.28}{99.19}$	$\underset{\pm 0.25}{99.27\cdot }$	$\underset{\pm 0.31}{99.18}$	$\underset{\pm 0.28}{99.19}$	$\underset{\pm 0.29}{99.18}$	$\underset{\pm 0.30}{99.18}$	$\underset{\pm 0.32}{99.16}$
kr-vs-kp	$\underset{\pm 0.02}{99.95}$	$\underset{\pm 0.01}{99.74}$	$\underset{\pm 0.02}{99.94}$	$\underset{\pm 0.01}{99.74}$	$\underset{\pm 0.01}{99.96\cdot }$	$\underset{\pm 0.01}{99.96\cdot }$	$\underset{\pm 0.01}{99.96\cdot }$	$\underset{\pm 0.01}{99.96\cdot }$
lymphography_bin	$\underset{\pm 1.45}{92.45}$	$\underset{\pm 1.09}{92.81}$	$\underset{\pm 1.10}{92.77}$	$\underset{\pm 1.23}{92.73}$	$\underset{\pm 1.33}{92.40}$	$\underset{\pm 1.25}{92.67}$	$\underset{\pm 1.13}{92.86}$	$\underset{\pm 1.16}{93.03\cdot }$
monks-1	$\underset{\pm 0.00}{100.00\cdot }$	$\underset{\pm 1.40}{91.52}$	$\underset{\pm 0.00}{100.00\cdot }$	$\underset{\pm 1.86}{87.75}$	$\underset{\pm 0.00}{100.00\cdot }$	$\underset{\pm 0.00}{100.00\cdot }$	$\underset{\pm 0.00}{100.00\cdot }$	$\underset{\pm 0.00}{100.00\cdot }$
primary-tumor	$\underset{\pm 0.61}{72.88}$	$\underset{\pm 0.56}{72.92}$	$\underset{\pm 0.79}{72.80}$	$\underset{\pm 0.58}{72.72}$	$\underset{\pm 0.54}{72.97\cdot }$	$\underset{\pm 0.51}{72.95}$	$\underset{\pm 0.82}{72.70}$	$\underset{\pm 0.61}{72.86}$
promoters	$\underset{\pm 1.06}{97.51}$	$\underset{\pm 0.92}{97.39}$	$\underset{\pm 0.87}{97.39}$	$\underset{\pm 0.87}{97.28}$	$\underset{\pm 1.09}{97.57\cdot }$	$\underset{\pm 1.13}{97.49}$	$\underset{\pm 0.89}{97.43}$	$\underset{\pm 0.88}{97.45}$
soybean	$\underset{\pm 0.08}{99.66}$	$\underset{\pm 0.09}{99.64}$	$\underset{\pm 0.09}{99.69\cdot }$	$\underset{\pm 0.07}{99.66}$	$\underset{\pm 0.10}{99.66}$	$\underset{\pm 0.09}{99.65}$	$\underset{\pm 0.07}{99.68}$	$\underset{\pm 0.06}{99.69\cdot }$
spect	$\underset{\pm 2.07}{83.60}$	$\underset{\pm 2.08}{83.58}$	$\underset{\pm 1.98}{83.79}$	$\underset{\pm 2.06}{83.75}$	$\underset{\pm 2.04}{83.61}$	$\underset{\pm 2.04}{83.63}$	$\underset{\pm 2.00}{83.93\cdot }$	$\underset{\pm 2.08}{83.81}$
splice	$\underset{\pm 0.04}{99.35\cdot }$	$\underset{\pm 0.04}{99.20}$	$\underset{\pm 0.02}{99.28}$	$\underset{\pm 0.04}{99.19}$	$\underset{\pm 0.03}{99.35\cdot }$	$\underset{\pm 0.04}{99.35\cdot }$	$\underset{\pm 0.02}{99.29}$	$\underset{\pm 0.02}{99.29}$
tic-tac-toe	$\underset{\pm 0.61}{98.03}$	$\underset{\pm 0.43}{97.86}$	$\underset{\pm 0.37}{98.25\cdot }$	$\underset{\pm 0.37}{98.25\cdot }$	$\underset{\pm 0.60}{98.03}$	$\underset{\pm 0.61}{98.03}$	$\underset{\pm 0.37}{98.25\cdot }$	$\underset{\pm 0.37}{98.25\cdot }$
zoo	$\underset{\pm 0.00}{100.00\cdot }$	$\underset{\pm 0.00}{100.00\cdot }$	$\underset{\pm 0.00}{100.00\cdot }$	$\underset{\pm 0.00}{100.00\cdot }$	$\underset{\pm 0.00}{100.00\cdot }$	$\underset{\pm 0.00}{100.00\cdot }$	$\underset{\pm 0.00}{100.00\cdot }$	$\underset{\pm 0.00}{100.00\cdot }$
**Rank**	4.38	5.13	3.06	5.44	4.00	3.88	**2.88**	3.13

**Table 4:** AUC performances on benchmark datasets. For each dataset the best performing kernel is highlighted with both the **boldface** font and with a dot (·).

Dataset	Linear	RBF	DNF [3]	d-mDNF	Tanimoto	σ-mDNF	avg.mDNF	avg.mCNF	avg.DNF	avg.CNF
breast	$\underset{\pm 0.91}{70.69}$	$\underset{\pm 1.63}{71.21}$	$\underset{\pm 1.55}{71.56}$	$\underset{\pm 1.50}{71.82}$	$\underset{\pm 1.99}{72.32\cdot }$	$\underset{\pm 1.77}{71.24}$	$\underset{\pm 1.69}{70.09}$	$\underset{\pm 1.85}{72.16}$	$\underset{\pm 1.97}{72.18}$	$\underset{\pm 1.73}{72.03}$
car_bin	$\underset{\pm 0.13}{98.98}$	$\underset{\pm 0.07}{99.96}$	$\underset{\pm 0.00}{100.00\cdot }$	$\underset{\pm 0.00}{100.00\cdot }$	$\underset{\pm 0.00}{100.00\cdot }$	$\underset{\pm 0.00}{100.00\cdot }$	$\underset{\pm 0.04}{99.82}$	$\underset{\pm 0.00}{100.00\cdot }$	$\underset{\pm 0.00}{100.00\cdot }$	$\underset{\pm 0.00}{100.00\cdot }$
crx	$\underset{\pm 0.41}{91.08}$	$\underset{\pm 0.36}{92.03}$	$\underset{\pm 0.21}{91.96}$	$\underset{\pm 0.23}{91.82}$	$\underset{\pm 0.26}{92.14\cdot }$	$\underset{\pm 0.36}{91.99}$	$\underset{\pm 0.21}{91.78}$	$\underset{\pm 0.30}{91.96}$	$\underset{\pm 0.27}{92.02}$	$\underset{\pm 0.25}{92.09}$
dna_bin	$\underset{\pm 0.10}{98.21}$	$\underset{\pm 0.06}{98.84}$	$\underset{\pm 0.19}{97.25}$	$\underset{\pm 0.05}{98.50}$	$\underset{\pm 0.05}{98.92}$	$\underset{\pm 0.06}{98.52}$	$\underset{\pm 0.05}{98.51}$	$\underset{\pm 0.05}{99.04}$	$\underset{\pm 0.05}{99.07}$	$\underset{\pm 0.05}{99.07\cdot }$
flare	$\underset{\pm 0.14}{94.85}$	$\underset{\pm 0.14}{94.87\cdot }$	$\underset{\pm 0.18}{93.72}$	$\underset{\pm 0.12}{94.53}$	$\underset{\pm 0.17}{94.84}$	$\underset{\pm 0.10}{94.82}$	$\underset{\pm 0.17}{93.89}$	$\underset{\pm 0.12}{94.76}$	$\underset{\pm 0.18}{94.76}$	$\underset{\pm 0.16}{94.86}$
house-v.	$\underset{\pm 0.18}{99.38\cdot }$	$\underset{\pm 0.17}{99.36}$	$\underset{\pm 0.24}{98.67}$	$\underset{\pm 0.26}{98.96}$	$\underset{\pm 0.26}{99.26}$	$\underset{\pm 0.21}{99.16}$	$\underset{\pm 0.23}{98.85}$	$\underset{\pm 0.20}{98.87}$	$\underset{\pm 0.25}{98.94}$	$\underset{\pm 0.25}{98.94}$
kr-vs-kp	$\underset{\pm 0.01}{99.14}$	$\underset{\pm 0.01}{99.98\cdot }$	$\underset{\pm 0.01}{99.94}$	$\underset{\pm 0.01}{99.97}$	$\underset{\pm 0.01}{99.94}$	$\underset{\pm 0.01}{99.98\cdot }$	$\underset{\pm 0.01}{99.97}$	$\underset{\pm 0.01}{99.94}$	$\underset{\pm 0.01}{99.97}$	$\underset{\pm 0.01}{99.94}$
lympho.	$\underset{\pm 1.20}{92.37}$	$\underset{\pm 1.28}{92.62}$	$\underset{\pm 0.82}{92.20}$	$\underset{\pm 1.01}{93.08}$	$\underset{\pm 1.10}{93.03}$	$\underset{\pm 1.17}{93.10}$	$\underset{\pm 0.89}{92.86}$	$\underset{\pm 1.02}{93.07}$	$\underset{\pm 1.01}{93.13}$	$\underset{\pm 1.08}{93.34\cdot }$
monks-1	$\underset{\pm 3.12}{46.32}$	$\underset{\pm 0.47}{99.10}$	$\underset{\pm 1.52}{89.87}$	$\underset{\pm 1.35}{91.98}$	$\underset{\pm 0.23}{99.70}$	$\underset{\pm 0.32}{99.71}$	$\underset{\pm 0.00}{100.00\cdot }$	$\underset{\pm 0.00}{100.00\cdot }$	$\underset{\pm 0.00}{100.00\cdot }$	$\underset{\pm 0.00}{100.00\cdot }$
primary-t.	$\underset{\pm 0.81}{73.31}$	$\underset{\pm 0.90}{72.75}$	$\underset{\pm 0.89}{73.01}$	$\underset{\pm 0.39}{73.41\cdot }$	$\underset{\pm 0.68}{73.00}$	$\underset{\pm 0.54}{72.99}$	$\underset{\pm 0.55}{73.37}$	$\underset{\pm 0.54}{73.06}$	$\underset{\pm 0.45}{73.12}$	$\underset{\pm 0.45}{73.34}$
promoters	$\underset{\pm 0.98}{97.23}$	$\underset{\pm 0.98}{97.37}$	$\underset{\pm 0.88}{95.07}$	$\underset{\pm 0.82}{97.65\cdot }$	$\underset{\pm 0.94}{97.31}$	$\underset{\pm 0.92}{97.29}$	$\underset{\pm 0.75}{97.61}$	$\underset{\pm 0.91}{97.46}$	$\underset{\pm 0.92}{97.37}$	$\underset{\pm 0.83}{97.41}$
soybean	$\underset{\pm 0.12}{99.47}$	$\underset{\pm 0.10}{99.68}$	$\underset{\pm 0.08}{99.56}$	$\underset{\pm 0.07}{99.72}$	$\underset{\pm 0.06}{99.65}$	$\underset{\pm 0.10}{99.74}$	$\underset{\pm 0.09}{99.73\cdot }$	$\underset{\pm 0.07}{99.72}$	$\underset{\pm 0.09}{99.71}$	$\underset{\pm 0.09}{99.71}$
spect	$\underset{\pm 1.82}{83.81\cdot }$	$\underset{\pm 1.82}{83.70}$	$\underset{\pm 1.90}{78.57}$	$\underset{\pm 1.94}{82.34}$	$\underset{\pm 1.92}{84.14}$	$\underset{\pm 1.77}{83.43}$	$\underset{\pm 1.97}{82.27}$	$\underset{\pm 2.04}{82.25}$	$\underset{\pm 1.99}{82.53}$	$\underset{\pm 1.95}{82.42}$
splice	$\underset{\pm 0.03}{98.48}$	$\underset{\pm 0.04}{99.12}$	$\underset{\pm 0.13}{98.27}$	$\underset{\pm 0.04}{99.31\cdot }$	$\underset{\pm 0.04}{99.11}$	$\underset{\pm 0.05}{99.01}$	$\underset{\pm 0.04}{99.30}$	$\underset{\pm 0.03}{99.25}$	$\underset{\pm 0.03}{99.29}$	$\underset{\pm 0.03}{99.28}$
t-t-t	$\underset{\pm 0.43}{97.86}$	$\underset{\pm 0.18}{98.53}$	$\underset{\pm 0.03}{99.94}$	$\underset{\pm 0.02}{99.97\cdot }$	$\underset{\pm 0.05}{99.89}$	$\underset{\pm 0.05}{99.88}$	$\underset{\pm 0.03}{99.96}$	$\underset{\pm 0.03}{99.95}$	$\underset{\pm 0.03}{99.96}$	$\underset{\pm 0.02}{99.95}$
zoo	$\underset{\pm 0.00}{100.00\cdot }$	$\underset{\pm 0.00}{100.00\cdot }$	$\underset{\pm 0.00}{100.00\cdot }$	$\underset{\pm 0.00}{100.00\cdot }$	$\underset{\pm 0.00}{100.00\cdot }$	$\underset{\pm 0.00}{100.00\cdot }$	$\underset{\pm 0.00}{100.00\cdot }$	$\underset{\pm 0.00}{100.00\cdot }$	$\underset{\pm 0.00}{100.00\cdot }$	$\underset{\pm 0.00}{100.00\cdot }$
**Rank**	7.25	5.25	7.81	4.13	5.00	5.13	5.31	4.56	3.50	**3.44**
